# The National Pension Fund

**Published:** 1888-03-10

**Authors:** 


### THE NATIONAL PENSION FUND FOR NURSES.

*Established October*, 1887. *Incorporated February*, 1888.

THIS fund is progressing rapidly. In addition to the £20,000 already invested with the Court of Chancery in the names of the trustees, Lord Rothschild, Messrs. Henry Hucks Gibbs, E. A. Hambro, and Junius S. Morgan, as security for the members and policy holders, some £6,000 has already been promised to start the Bonus Fund. It will be the aim of the Council to secure, to each nurse who is willing to save at least one-eighth of her wages per annum, a sick allowance of ten shillings per week when totally incapacitated by illness, with a pension at 60 of at least £26 per annum for the rest of her life. The General Purposes Committee have had Mr. Burdett's draft prospectus under consideration during the week, and the Council has been summoned to finally approve it. There is therefore no reason why those who wish to join the National Pension Fund should have to wait many days longer before doing so. It remains to be seen how many of the 15,000 nurses will fail to join when it is realised that every nurse may hope to secure at sixty years of age two sovereigns per annum in pension, in addition to every three she has subscribed for out of her own savings? The Bank of England has undertaken with its branches to be the bankers of the fund, and anyone who is desirous of having any point of difficulty cleared up should call upon the Secretary, Mr. Philip Grove, at the office, 38, Old Jewry, London, E.C., on any week day, except Saturday, between ten and four o'clock. Next week we propose to publish the objects of the National Pension Fund for Nurses as set forth in the Memorandum and Articles of Association, copies of which may be obtained from the Secretary on application on payment of one shilling in stamps.

